# Detection of unknown ototoxic adverse drug reactions: an electronic healthcare record-based longitudinal nationwide cohort analysis

**DOI:** 10.1038/s41598-021-93522-z

**Published:** 2021-07-07

**Authors:** Suehyun Lee, Jaehun Cha, Jong-Yeup Kim, Gil Myeong Son, Dong-Kyu Kim

**Affiliations:** 1 Department of Biomedical Informatics, College of Medicine, Konyang University, Daejeon, Republic of Korea; 2 Department of Otorhinolaryngology-Head and Neck Surgery, College of Medicine, Konyang University, Daejeon, Republic of Korea; 3 Department of Otorhinolaryngology-Head and Neck Surgery, Chuncheon Sacred Heart Hospital, Hallym University College of Medicine, 77, Sakju-ro, Chuncheon-si, Gangwon-do 24253 Republic of Korea; 4 Division of Big Data and Artificial Intelligence, Chuncheon Sacred Heart Hospital, Hallym University College of Medicine, Chuncheon, Republic of Korea

**Keywords:** Diseases, Health care, Medical research, Risk factors

## Abstract

Ototoxic medications can lead to significant morbidity. Thus, pre-marketing clinical trials have assessed new drugs that have ototoxic potential. Nevertheless, several ototoxic side effects of drugs may remain undetected. Hence, we sought to retrospectively investigate the potential risk of ototoxic adverse drug reactions among commonly used drugs via a longitudinal cohort study. An electronic health records-based data analysis with a propensity-matched comparator group was carried out. This study was conducted using the MetaNurse algorithm for standard nursing statements on electronic healthcare records and the National Sample Cohort obtained from the South Korea National Health Insurance Service. Five target drugs capable of causing ototoxic adverse drug reactions were identified using MetaNurse; two drugs were excluded after database-based analysis because of the absence of bilateral hearing loss events in patients. Survival analysis, log-rank test, and Cox proportional hazards regression models were used to calculate the incidence, survival rate, and hazard ratio of bilateral hearing loss among patients who were prescribed candidate ototoxic drugs. The adjusted hazard ratio of bilateral hearing loss was 1.31 (1.03–1.68), 2.20 (1.05–4.60), and 2.26 (1.18–4.33) in cimetidine, hydroxyzine, and sucralfate users, respectively. Our results indicated that hydroxyzine and sucralfate may cause ototoxic adverse drug reactions in patients. Thus, clinicians should consider avoiding co-administration of these drugs with other well-confirmed ototoxic drugs should be emphasized.

## Introduction

Drug-induced ototoxicity is defined as a functional impairment, such as hearing and/or balance disorders, depending on the involvement of the cochlear and/or vestibular system, respectively, or both^1–3^, characterized by temporary or permanent inner hair cell damage caused by therapeutic agents^1^. Almost all ototoxicity events spontaneously resolve after treatment discontinuation, although several events often result in permanent serious consequences, and detract from the patient’s quality of life^1,4^. Several monitoring protocols for drug-induced ototoxicity exist, although their practical use is questionable due to adherence issues^5–7^. Generally, pre-marketing studies of new pharmaceuticals are important to detect adverse drug reactions (ADR), although these studies sometimes cannot reliably detect ADR^8^. Post-marketing surveillance could facilitate a full understanding of the risk–benefits of drugs^9^. Thus, both pre-marketing clinical trials and post-marketing data are necessary to detect unknown information on ototoxicity.

To date, the commonest ototoxic drugs in clinical use include aminoglycoside antibiotics and platinum-based chemotherapeutic agents (cisplatin and carboplatin) which have well-documented efficacy against various infections and malignancies, respectively, in both children and adults^10^. The panorama of drug-induced ototoxicity has expanded in the last few decades from aminoglycoside antibiotics and platinum-based chemotherapeutic agents to include non-steroidal anti-inflammatory drugs, loop diuretics, and other antimicrobial or antineoplastic agents^11^. Several reports indicate that more than 600 drugs have ototoxic potential. Nevertheless, the ototoxic effects of several drugs may remain undetected. Thus, researching the possibility of drug-induced ototoxicity is meaningful in clinical fields^1,4,5,12^. Recently, we developed MetaNurse, an algorithm for detecting ADRs based on a meta-analysis technique normalized on a yearly basis and an improved patient-sampling and comparison group creation strategy, in an initial attempt to investigate candidate drugs that can possibly induce ototoxicity^13^. Therefore, in this study, we investigate the potential risk of ototoxic adverse drug reactions among commonly used drugs via the MetaNurse and the longitudinal cohort dataset.

## Results

### Search for drug-induced ototoxicity based on the MetaNurse algorithm

**Figure 1:**
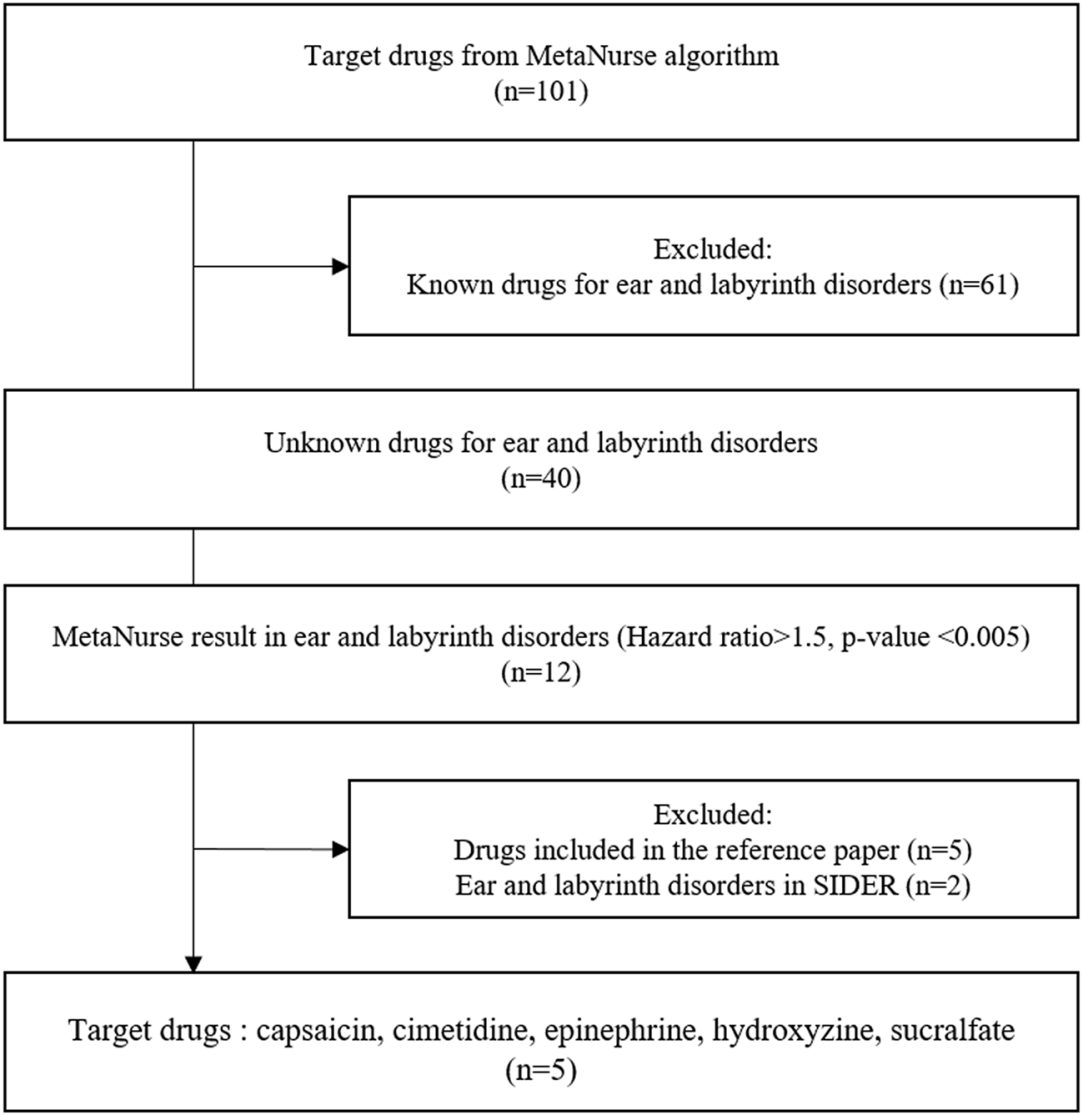
Process for target drug selection using the MetaNurse algorithm.

We first selected 101 target drugs based on the activity-enabled pharmacovigilance MetaNurse algorithms, including Beers criteria medications (n = 107), medicines with pediatric use restrictions (n = 79), and the United Nations list of banned drugs (n = 28) (Fig. 1). We excluded 61 drugs due to previously known ototoxic effects. Thus, we assessed 40 drugs that have significant associations with ear and labyrinth disorders, and only 12 of the drugs showed HR > 1.5 (*p* < 0.005) in MetaNurse. We extracted five target drugs (capsaicin, cimetidine, epinephrine, hydroxyzine, and sucralfate) after the exclusion process based on a review of the Side Effect Resource (SIDER 4.1) and ototoxicity-related articles.

### Incidence of subsequent bilateral hearing loss in patients who were prescribed candidate drugs

**Figure 2:**
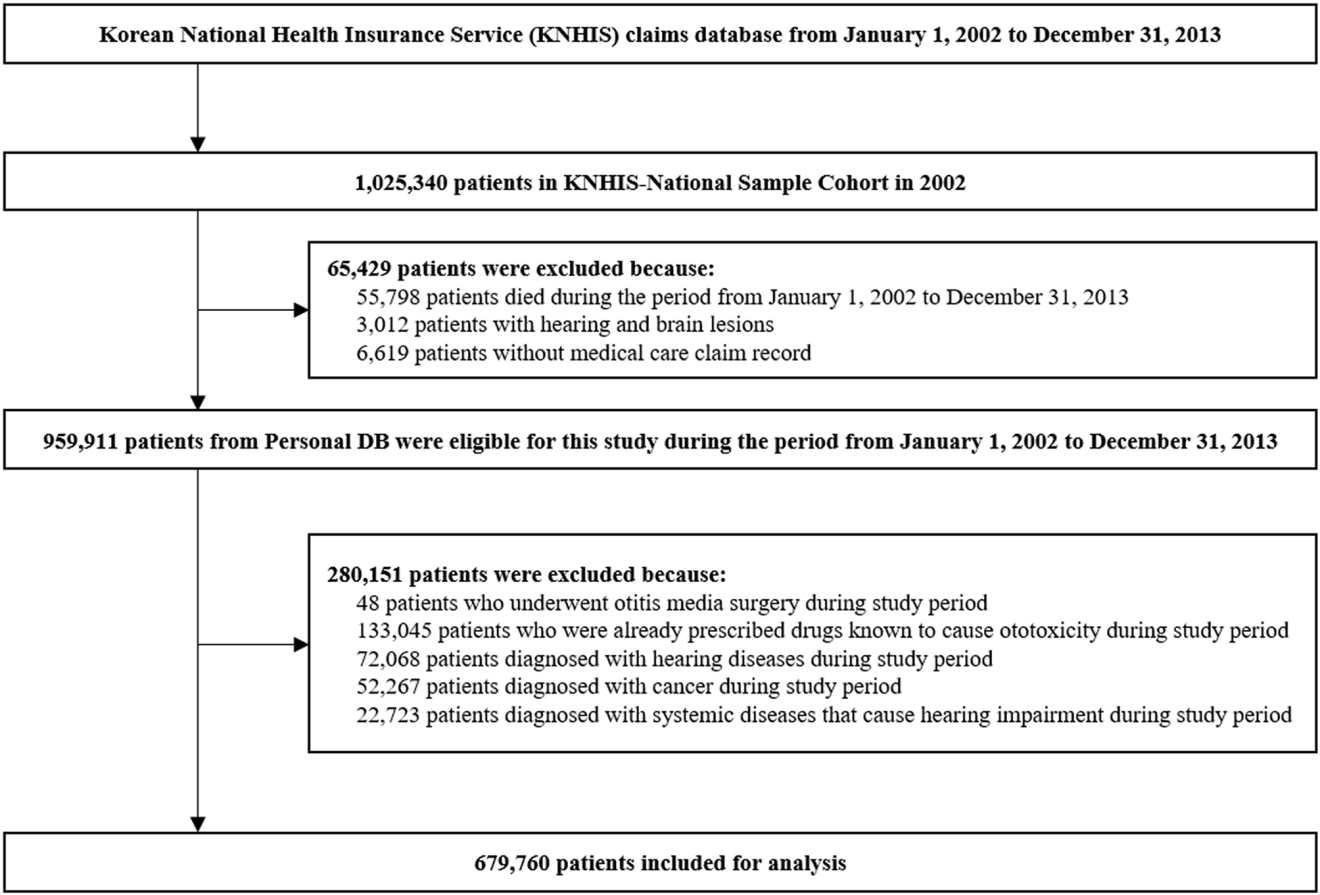
Selection of the study participants.

A total of five target drugs were investigated for the possibility of subsequent development of bilateral hearing loss based on the South Korea National Health Insurance Service–National Sample Cohort (KNHIS–NSC) database. Among the five target drugs, capsaicin and epinephrine were excluded from the final analysis because no bilateral hearing loss event had occurred until 2013. This cohort study included patients who were prescribed each candidate drug (i.e., cimetidine, hydroxyzine, and sucralfate) for more than 14 days during the index period (January 2002 to December 2013, Supplementary Fig. 1). A schematic description of the study flow is presented in Fig. 2. Each of the three cohorts (prescribed cimetidine, hydroxyzine, and sucralfate) showed similar distributions of sex, age, residential area, household income, and the CCI between the medication and non-medication groups because these variables were used for sample matching, which indicates that group matching was performed appropriately (Supplementary Tables 1–3).

**Figure 3:**
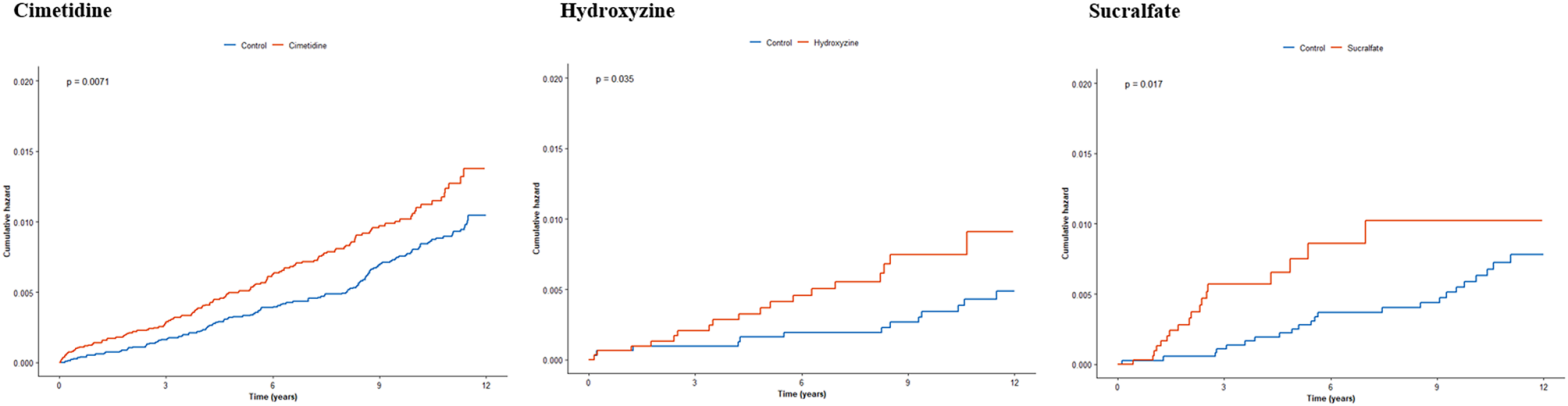
Kaplan–Meier survival curves and log-rank tests for bilateral hearing loss in patients who were prescribed cimetidine, hydroxyzine, and sucralfate.

In this study, the cimetidine non-users and users had a total of 155,039.9 and 122,629.5 person-years for bilateral hearing loss events. The hydroxyzine non-users and users had a total of 33,551.4 and 23,384.7 person-years for bilateral hearing loss events. Additionally, the sucralfate non-users and users had a total of 36,481.9 and 13,825.6 person-years for bilateral hearing loss events. We used univariate and multiple Cox regression models to analyze the HR for the development of bilateral hearing loss. After adjusting for sociodemographic and comorbidities factors, the cimetidine, hydroxyzine, and sucralfate users had an associated prospective bilateral hearing loss development with an adjusted HR of 1.31 (95% CI, 1.03–1.68), 2.20 (95% CI, 1.05–4.60), and 2.26 (95% CI, 1.18–4.33), respectively (Table 1). Kaplan–Meier survival curves with log-rank tests for the 12-year follow-up period are presented in Fig. 3, and indicate that patients who were prescribed cimetidine, hydroxyzine, and sucralfate developed the bilateral hearing loss more frequently than patients who were not prescribed those medicines.

**Table 1:** Incidence per 1000 person-years and HR (95% CIs) of bilateral hearing loss between non-drug user and drug user (cimetidine, hydroxyzine, and sucralfate).

Variables	N	Case	Person-years	Incidence	Unadjusted HR (95% CI)	Adjusted HR (95% CI)
**Cimetidine**						
Comparison group	15,494	128	155,039.9	0.83	1.00 (ref)	1.00 (ref)
Cimetidine user	15,494	133	122,629.5	1.08	1.40 (1.09–1.79)	1.31 (1.03–1.68)
**Hydroxyzine**						
Non-hydroxyzine user	3193	13	33,551.4	0.39	1.00 (ref)	1.00 (ref)
Hydroxyzine user	3193	18	23,384.7	0.77	2.16 (1.04–4.48)	2.20 (1.05–4.60)
**Sucralfate**						
Non-sucralfate	3750	23	36,481.9	0.63	1.00 (ref)	1.00 (ref)
Sucralfate user	3750	18	13,825.6	1.30	2.18 (1.14–4.18)	2.26 (1.18–4.33)

HR, hazard ratio; CI, confidence interval.

### External validation of the possibility for drug-induced ototoxicity with cimetidine, hydroxyzine, and sucralfate

To confirm our findings, we performed external validation using United States Food and Drug Administration Adverse Event Reporting System (FAERS) data from 2012 to 2018 (Table 2) wherein we quantified the total number of otologic ADR, including ototoxicity, deafness, and tinnitus. The PRR and ROR with 95% CI for hydroxyzine versus total drugs in otologic adverse events were 1.40 (1.11–1.76) and 1.40 (1.10–1.76), respectively. The PRR and ROR for sucralfate versus total drugs in otologic adverse events were 1.55 (1.04–2.32) and 1.56 (1.00–2.32), respectively. However, we could not find any significant PRR and ROR for cimetidine.

**Table 2:** External validation of ototoxicity signal in FDA adverse event reporting system database.

	Proportional reporting ratio	Reporting odds ratio
Cimetidine	1.48 (0.74–2.96)	1.49 (0.64–2.94)
Hydroxyzine	1.40 (1.11–1.76)	1.40 (1.10–1.76)
Sucralfate	1.55 (1.04–2.32)	1.56 (1.00–2.32)

## Discussion

Using MetaNurse and KNHIS–NSC, this study investigated certain drugs with the potential for causing drug-induced ototoxicity^13–17^. To our knowledge, this is the first study to evaluate the possibility of ototoxicity with the use of cimetidine, hydroxyzine, and sucralfate. We used MetaNurse as a post-marketing pharmacovigilance tool based on EHR and the South Korean nationwide population insurance data. We initially selected several candidate drugs for ototoxicity, and then conducted a retrospective cohort study to evaluate the risk of bilateral hearing loss in patients who were prescribed the candidate drugs. External validation was performed to verify the novel findings using the spontaneous reports FAERS database.

Ototoxicity is a pharmacological adverse reaction affecting the inner ear or auditory nerve, characterized by cochlear or vestibular dysfunction^1,2^. To date, aminoglycoside antibiotics and platinum-based chemotherapy, mainly cisplatin, are the most important ototoxic drugs that are commonly used in clinical practice^10^. These medications play an important role in modern medicine although they cause harmful effects and lead to significant patient morbidity. Thus, the early detection of ototoxicity through prospective monitoring is very important because it allows the consideration of treatment modifications to minimize or prevent permanent hearing loss and balance impairment^18–20^. However, some pharmacotherapy associated with the development of ototoxicity could remain undetected for a long time because physicians do not recognize the need for drug concentration monitoring for these drugs. Therefore, studies are needed to explore possible ototoxic ADR based on EHR^13,21–23^.

In this study, we detected capsaicin, cimetidine, epinephrine, hydroxyzine, and sucralfate as possible candidate ototoxic drugs using the MetaNurse algorithm, which is based on nurse statements because nurses reportedly play a more important role in discovering and spontaneously reporting ADR than doctors and pharmacists^24–27^. However, on a limitation is that the result of the MetaNurse algorithm extracts the candidate drug list as the result of analysis for one hospital. To overcome this issue, we further verified candidate drugs obtained from the MetaNurse algorithm through a nationwide cohort sample because this dataset could accurately represent the characterization of South Koreans. In the analysis of KNHIS–NSC data, we used the diagnostic codes of bilateral hearing loss (H90.0, H90.3, and H90.6). There is no diagnostic code for ototoxicity and, therefore, we used the diagnostic code for bilateral hearing loss, which is the commonest phenotype of ototoxicity. Interestingly, we observed an increased incidence of bilateral hearing loss in patients who received cimetidine, hydroxyzine, and sucralfate when compared with those in the propensity score-matched comparator group developed based on sociodemographic factors, comorbidities, and enrollment year. After the analysis of KNHIS–NSC data, external validation was performed and showed that the values of PRR and ROR in hydroxyzine and sucralfate were positive indicators of ototoxicity because their 95% CI values were statistically significant; however, there was no significance in the PRR and ROR of cimetidine. Collectively, this means that these novel study findings, which indicate that hydroxyzine and sucralfate have a potential ototoxic effect, may be reliable.

Hydroxyzine is a histamine receptor (H_1_) antagonist that has been widely used to treat allergic skin reactions, such as pruritus and urticaria, and it is also used as an anti‐emetic and anti‐cholinergic medication. Additionally, hydroxyzine can reduce central nervous system activity. Thus, in psychiatric disorders, hydroxyzine has been used widely as a sedative to relieve anxiety and tension in generalized anxiety disorder since the 1980s^28,29^. The commonest side effect of hydroxyzine is dry mouth because of the anticholinergic effect. Drowsiness and involuntary motor activity could also occur when patients take considerably higher than recommended doses. Drug allergic reactions, headaches, and hallucinations are rarely reported in post-marketing surveillance data, but we could not find any information on hydroxyzine-induced ototoxicity. Sucralfate has been commonly used to treat and prevent duodenal or stress ulcers because it adheres to damaged ulcer tissue and protects against acid and enzyme damage, leading to better healing. Treatment with other medications, such as antibiotics, may be needed to treat and prevent the ulcer recurrence caused by some types of bacteria (e.g., *H. pylori*)^30^. Sucralfate can sometimes cause some side effects, including constipation, hives, rash, itching, difficulty in breathing or swallowing, and angioedema. Post-marketing surveillance reports revealed hypersensitivity reactions and hyperglycemia as side effects, but there were no reports of sucralfate-related ototoxicity.

This study has several limitations. First, to date, mechanisms of drug-induced ototoxicity with hydroxyzine and sucralfate have not been defined previously. Thus, further mechanistic studies are needed to determine their ototoxic effects. Second, we used bilateral hearing loss as a diagnostic code; however, in some cases, bilateral hearing loss may have developed due to non-ototoxicity-related causes. However, ototoxicity is the commonest cause of bilateral hearing loss, which suggests that this issue may be an acceptable limitation. Third, data on the degree of hearing impairment were not available in the registry (KNHIS–NSC); therefore, we could not ascertain the degree of hearing difficulty effected by the candidate drug and whether that side effect was transient or permanent. Fourth, the drug dosage, frequency, the exact duration of the prescription, and compliance of the participants also could affect the occurrence of drug-induced ototoxicity. However, in this study, we could not access that information. Fifth, we could not access other health data, including body mass index, lipid profile, and information on behavioral risk factors, such as smoking or alcohol consumption. Therefore, these possible confounding factors could not be controlled in the study analyses. Sixth, drugs related to vestibulopathy symptoms such as tinnitus or vertigo could happen in daily clinical practice, but we could not involve these events in this study because this issue is very difficult to define by diagnostic code. Finally, some bilateral hearing loss events in this study might be caused by other drugs not part of the analyzed candidate drugs (101 drugs). Thus, this issue could influence distortedly the causality relationship in the present study.

This study investigated the detection of unknown ototoxic drugs among widely used drugs using MetaNurse and the KNHIS–NSC. We found that patients who were prescribed cimetidine, hydroxyzine, and sucralfate had a significant risk of bilateral hearing loss events. Additionally, the possibility of ototoxicity with hydroxyzine and sucralfate drugs was confirmed by an external validation based on FAERS. This study provides new insight into the potential risk of ototoxic ADR with hydroxyzine and sucralfate. Further studies are needed to elucidate the underlying pathophysiological mechanisms mediating the drug-related ototoxicity identified for these three drugs.

## Methods

This study was approved by the Institutional Review Board of Hallym Medical University Chuncheon Sacred Hospital (IRB No. 2016-05-052). The requirement for written informed consent was waived by the approving authority because the KNHIS–NSC dataset comprises de-identified secondary data for research purposes. All methods were carried out in accordance with relevant guidelines and regulations.

### MetaNurse

MetaNurse is an electronic healthcare record (EHR)-based pharmacovigilance algorithm applied to determine the frequency of standard nursing statements (SNS) or ADR symptoms^13^ and to quantify symptoms only detected through bedside nurse observations that cannot be observed in a laboratory. Based on SNS in EHR, we identified the patients who experienced ADR and determined which drugs to include in a safety review. MetaNurse comprises a meta-analysis technique that is normalized annually with improved patient-sampling and comparison-group creation. In MetaNurse, patients who were unexposed to the drug were defined as the comparator group, and ADR that were recorded more than twice in SNS after the initial drug administration constituted the signal. Hazard ratios (HR) were used as statistical indicators that were subsequently computed by adjusting age, sex, department, and disease severity. We analyzed all inpatient EHR data that were obtained from January 1, 2005 to December 31, 2011.

### Korean National Health Insurance Service

South Korea has maintained a nationwide health insurance system since 1963. Nearly all of the national health system data are centrally stored in large databases of the Korean National Health Insurance Service (KNHIS). The KNHIS controls all medical costs across seven beneficiaries, medical providers, and the government. The KNHIS database uses the Korean Classification of Diseases, which is very similar to the International Classification of Diseases, for diagnostic practice codes and includes nearly all medical data, including diagnostic codes, procedures, prescriptions, and personal information. No patient healthcare records are duplicated or omitted because all South Korean residents receive a unique identification number at birth. This study used a representative sample from KNHIS–NSC database. Data from 2002 to 2013 were collected and yielded 1,025,340 nationally representative and randomly selected subjects (approximately 2.2% of the South Korean population in 2002)^31,32^. Stratified random sampling was performed using 1476 strata and by age (18 groups), sex (2 groups), and income level (41 groups, including 40 health insurance beneficiaries and 1 medical aid) among the South Korean population (46 million in 2002)^15,16^.

### Study population

The patient groups for this study included all patients who were prescribed the MetaNurse-proposed candidate ototoxic drugs between January 2002 and December 2013. Each patient was tracked until 2013, and patients diagnosed with bilateral hearing loss (H90.0, H90.3, and H90.6) were identified. We excluded the following patients: (1) those diagnosed with sudden sensorineural hearing loss, Ménière’s disease, vestibulitis, and acoustic neuroma; (2) those who underwent otologic surgeries; (3) those who died of any causes between 2002 and 2013; (4) those diagnosed with any malignancy; (5) those diagnosed with hearing and brain lesion disability; and (6) those diagnosed with other systemic diseases related to hearing impairment (E703, Q878, H905, A770, A692, G35, G932, I650, C880, D891, D571, Z951, G468, H518, M300, M9419, M313, and B24). Moreover, we excluded patients who were prescribed several drugs having potential ototoxic effects (aspirin/acetylsalicylic acid, aminoglycosides, interferon, cisplatin, cyclosporine, vinblastine, vincristine, gentamicin, tobramycin, amikacin, streptomycin, carboplatin, ibuprofen, naproxen, furosemide, and torasemide).

### Predictive and outcome variables

Details of the patients’ age, sex, residence, household income, and Charlson Comorbidity Index (CCI) were obtained from the database. The study population was divided into three age groups (< 45, 45–64, and > 65 years), three income groups (low, middle, and high as ≤ 30%, 30.1–69.9%, and ≥ 70% of the median, respectively), three residential areas (Seoul, the largest metropolitan region in South Korea; other metropolitan South Korean cities; and small cities and rural areas), and three CCI groups (0, 1, and ≥ 2). The CCI is a widely used weighted composite index for comorbidities, such as myocardial infarction, congestive heart failure, peripheral vascular disease or bypass, previous cerebrovascular accident or transient ischemic attack, dementia, chronic pulmonary disease, gastrointestinal ulcer disease, liver disease, moderate or severe renal disease, connective tissue disease or rheumatic disease, diabetes, diabetes with end-organ damage, acquired immune deficiency syndrome, non-metastatic solid tumor, leukemia, lymphoma, multiple myeloma, and metastatic tumor. The study endpoint was the incidence of bilateral hearing loss. All patients who had no events and were alive until December 31, 2013 were censored after this time point. In this longitudinal study, we conducted 1:1 propensity score matching according to sociodemographic factors. In the comparator group, we matched the enrollment year of subjects as the prescribed year of the respective case in the patient group. Collectively, the risks of bilateral hearing loss were compared between the patient and comparators groups using person-years at risk, which was defined as the duration between either the initiation of ototoxic drug use and the initial date of diagnosis of bilateral hearing loss or the end point (December 31, 2013).

### External validation

To verify the novel study findings, we used the publicly available FAERS database, which is a spontaneous reporting system that contains data on adverse events and medication errors, to identify the association between candidate drugs and adverse events by the case/non-case method. All reports that were published in the FAERS database from January 2012 to December 2018 were accessed from the official website of the U.S. Food and Drug Administration (http://www.fda.gov/)^33^.

### Statistical analyses

We employed 1:1 propensity score matching according to age, sex, residential area, household income, and the CCI. Incidence rates per 1000 person-years for bilateral hearing loss were obtained by dividing the number of patients with bilateral hearing loss by the person-years at risk. The overall disease-free survival rate was determined using Kaplan–Meier survival curves for the entire observation period. To identify whether the candidate drugs increased the risk of bilateral hearing loss, we used Cox proportional hazard regression to calculate the hazard ratio (HR) and 95% confidence intervals (CI), after adjusting for other predictor variables. For external validation, a case/non-case analysis was performed by calculating the proportional reporting ratio (PRR) and the reporting odds ratio (ROR) with 95% CI. All statistical analyses were performed using R version 3.6.0 (R Foundation for Statistical Computing, Vienna, Austria) with a significance level of 0.05.

## Supplementary Information

Supplementary Figure Legend.

Supplementary Figure.

Supplementary Tables.

## Data Availability

The datasets generated and/or analyzed during the current study are not publicly available due to the policy of Korea National Health Insurance Service but are available from the corresponding author on reasonable request. The corresponding author had full access to all the data in the study and takes responsibility for the integrity of the data and the accuracy of the data analysis.
