# Supplementary figures and images for: Detection of unknown ototoxic adverse drug reactions: an electronic healthcare record-based longitudinal nationwide cohort analysis
Source: Sci Rep. 2021 Jul 7;11:14045. doi: 10.1038/s41598-021-93522-z (PMC8263785; doi:10.1038/s41598-021-93522-z)

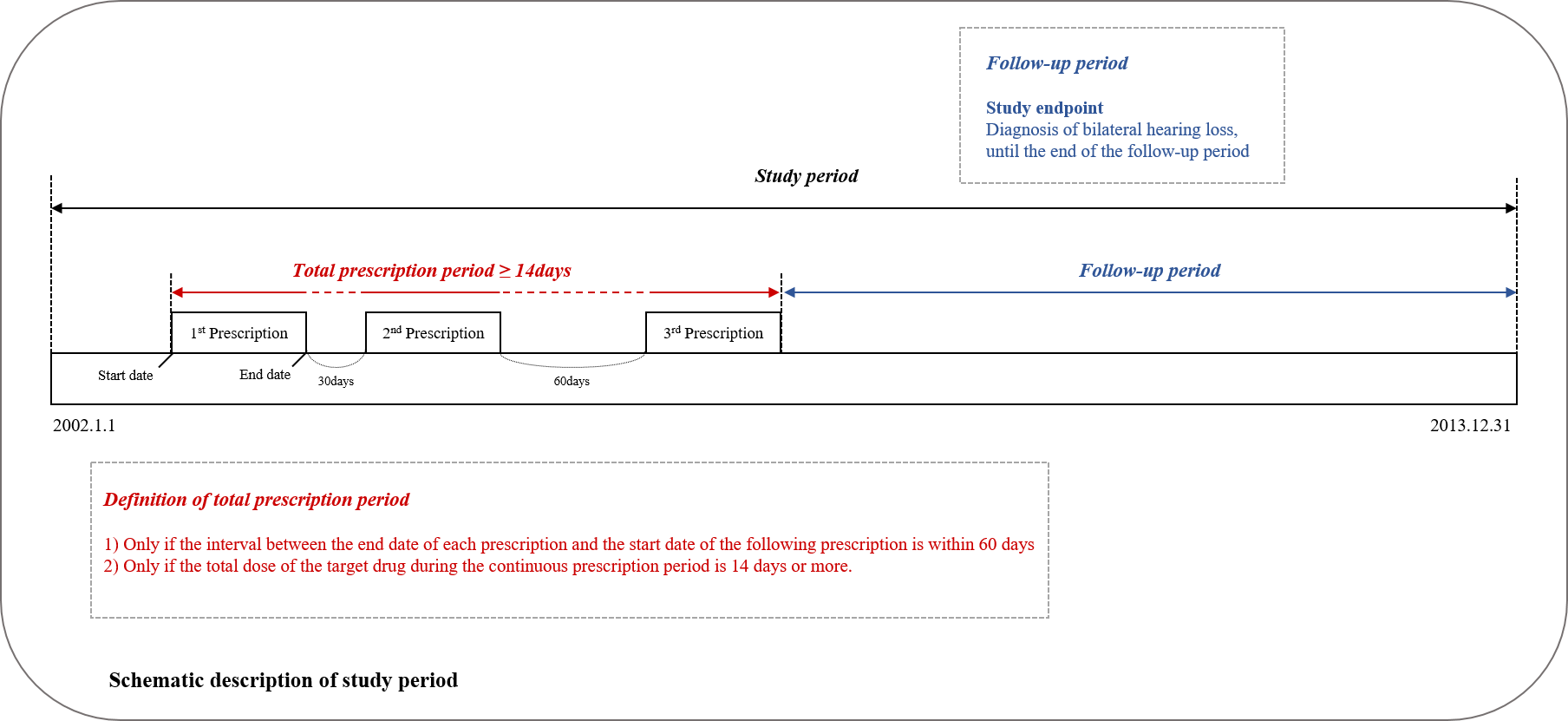

Supplement: Supplementary file 2 — Supplementary Figure. [file 41598_2021_93522_MOESM2_ESM.tif]
