# Supplementary material for: Detection of unknown ototoxic adverse drug reactions: an electronic healthcare record-based longitudinal nationwide cohort analysis
Source: Sci Rep. 2021 Jul 7;11:14045. doi: 10.1038/s41598-021-93522-z (PMC8263785; doi:10.1038/s41598-021-93522-z)
Supplement: Supplementary file 3 — Supplementary Tables. [file 41598_2021_93522_MOESM3_ESM.docx]

**Detection of Unknown Ototoxic Adverse Drug Reactions: An Electronic Healthcare Record-based Longitudinal Nationwide Cohort Analysis**

Suehyun Lee, Ph.D, Jaehun Cha, B.S, Jong-Yeup Kim, M.D, Ph.D, Gil Myeong Son, M.D, and Dong-Kyu Kim, M.D, Ph.D

**Supplementary table 1.** Characteristics of the study subjects for cimetidine cohort

| **Variables** | **Comparison (n=15,494)** | **Cimetidine user (n=15,494)** |
| --- | --- | --- |
| **Sex** |  |  |
| *Male* | 8,574(55.3%) | 7,922 (51.1%) |
| *Female* | 6,920 (44.7%) | 7,572 (48.9%) |
| **Ages (years)** |  |  |
| *<45* | 8,897 (57.4%) | 8,812 (56.9%) |
| *45-64* | 4,963 (32.0%) | 5,106 (33.0%) |
| *>64* | 1,634 (10.5%) | 1,576 (10.2%) |
| **Residence** |  |  |
| *Seoul (metropolitan)* | 4,083 (26.4%) | 3,800 (24.5%) |
| *2^nd^ area (other metropolitan)* | 3,786 (24.4%) | 3,954 (25.5%) |
| *3^rd^ area* | 7,625 (49.2%) | 7,740 (50.0%) |
| **Household income** |  |  |
| *Low (0-30%)* | 3,480 (22.5%) | 3,479 (22.5%) |
| *Middle (30-70%)* | 5,719 (36.9%) | 5,915 (38.2%) |
| *High (70-100%)* | 6,295 (40.6%) | 6,100 (39.4%) |
| **CCI** |  |  |
| *0* | 2,295 (14.8%) | 2,161 (13.9%) |
| *1* | 3,903 (25.2%) | 3,820 (24.7%) |
| *2* | 9,296 (60.0%) | 9,513 (61.4%) |

**Supplementary table 2.** Characteristics of the study subjects for hydroxyzine cohort

| **Variables** | **Comparison (n=3,193)** | **Hydroxyzine user (n=3,193)** |
| --- | --- | --- |
| **Sex** |  |  |
| *Male* | 1,905 (59.7%) | 1,870 (58.6%) |
| *Female* | 1,288 (40.3%) | 1,323 (41.4%) |
| **Ages (years)** |  |  |
| *<45* | 2,637 (82.6%) | 2,566 (80.4%) |
| *45-64* | 467 (14.6%) | 495 (15.5%) |
| *>64* | 89 (2.8%) | 132 (4.1%) |
| **Residence** |  |  |
| *Seoul (metropolitan)* | 857 (26.8%) | 846 (26.5%) |
| *2^nd^ area (other metropolitan)* | 774 (24.2%) | 782 (24.5%) |
| *3^rd^ area* | 1,562 (48.9%) | 1,565 (49.0%) |
| **Household income** |  |  |
| *Low (0-30%)* | 557 (17.4%) | 585 (18.3%) |
| *Middle (30-70%)* | 1,351 (42.3%) | 1,346 (42.2%) |
| *High (70-100%)* | 1,285 (40.2%) | 1,262 (39.5%) |
| **CCI** |  |  |
| *0* | 773 (24.2%) | 730 (22.9%) |
| *1* | 1,498 (46.9%) | 1,500 (47.0%) |
| *2* | 922 (28.9%) | 963 (30.2%) |

**Supplementary table 3.** Characteristics of the study subjects for sucralfate cohort

| **Variables** | **Comparison (n=3,750)** | **Sucralfate user (n=3,750)** |
| --- | --- | --- |
| **Sex** |  |  |
| *Male* | 2,426 (64.7%) | 2,392 (63.8%) |
| *Female* | 1,324 (35.3%) | 1,358 (36.2%) |
| **Ages (years)** |  |  |
| *<45* | 2,215 (59.1%) | 2,237 (59.7%) |
| *45-64* | 1,165 (31.1%) | 1,209 (32.2%) |
| *>64* | 370 (9.9%) | 304 (8.1%) |
| **Residence** |  |  |
| *Seoul (metropolitan)* | 830 (22.1%) | 780 (20.8%) |
| *2^nd^ area (other metropolitan)* | 1,058 (28.2%) | 1,101 (29.4%) |
| *3^rd^ area* | 1,862 (49.7%) | 1,869 (49.8%) |
| **Household income** |  |  |
| *Low (0-30%)* | 1,061 (28.3%) | 1,104 (29.4%) |
| *Middle (30-70%)* | 1,345 (35.9%) | 1,365 (36.4%) |
| *High (70-100%)* | 1,344 (35.8%) | 1,281 (34.2%) |
| **CCI** |  |  |
| *0* | 708 (18.9%) | 682 (18.2%) |
| *1* | 1,053 (28.1%) | 1,028 (27.4%) |
| *2* | 1,989 (53.0%) | 2,040 (54.4%) |
